# Food Frequency Questionnaire Personalisation Using Multi-Target Regression

**DOI:** 10.3390/nu14193943

**Published:** 2022-09-23

**Authors:** Nina Reščič, Oscar Mayora, Claudio Eccher, Mitja Luštrek

**Affiliations:** 1 Department of Intelligent Systems, Jožef Stefan Institute, 1000 Ljubljana, Slovenia; 2 Jožef Stefan International Postgraduate School, 1000 Ljubljana, Slovenia; 3 Fondazione Bruno Kessler, 38123 Trento, Italy

**Keywords:** Food Frequency Questionnaires, dietary assessment, self-monitoring, machine learning, multi-target regression, feature selection

## Abstract

Fondazione Bruno Kessler is developing a mobile app prototype for empowering citizens to improve their health conditions through different lifestyle interventions that will be incorporated into a mobile application for lifestyle promotion of the Province of Trento in the context of the Trentino Salute 4.0 Competence Center. The envisioned interventions are based on promoting behaviour change in various domains such as physical activity, mental health and nutrition. In particular, the nutrition component is a self-monitoring module that collects dietary habits to analyse them and recommend healthier eating behaviours. Dietary assessment is completed using a Food Frequency Questionnaire on the Mediterranean diet that is presented to the user as a grid of images. The questionnaire returns feedback on 11 aspects of nutrition. Although the questionnaire used in the application only consists of 24 questions, it still could be a bit overwhelming and a bit crowded when shown on the screen. In this paper, we tried to find a machine-learning-based solution to reduce the number of questions in the questionnaire. We proposed a method that uses the user’s previous answers as additional information to find the goals that need more attention. We compared this method with a case where the subset of questions is randomly selected and with a case where the subset is chosen using feature selection. We also explored how large the subset should be to obtain good predictions. All the experiments are conducted as a multi-target regression problem, which means several goals are predicted simultaneously. The proposed method adjusts well to the user in question and has the slightest error when predicting the goals.

## 1. Introduction

Food Frequency Questionnaires (FFQs) are inexpensive and probably one of the most commonly used dietary tools for self-assessment. Therefore, several applications incorporate FFQs to monitor dietary habits [1,2]. For example, Fondazione Bruno Kessler developed the Lifestyle Behaviour Change (LBC) application to offer people from the Trentino region an application to self-monitor several aspects of a healthy lifestyle, with nutrition habits being one of them.

The FFQ in the LBC application is based on a Mediterranean diet, which has been shown to have a wide range of benefits for health [3], for instance, preventing chronic diseases [4,5]. The FFQ used in the LBC application consists of 24 questions and returns feedback on 11 aspects of nutrition, which users can set as goals to follow. The questionnaire is presented innovatively as a grid of images where the users self-report the number of consumed portions of a particular food group by pushing the respective button. Although the questionnaire is relatively short, it can still be hard to read on smaller screens or for elderly users. Since the users can select goals they would like to monitor more deliberately, the application’s questions on the screen could be reduced by adapting to these goals.

Although performing well, most automated approaches for generating or adapting FFQs rely heavily on expert knowledge. For instance, Molag et al. [6] developed a data-based computer system that can generate an FFQ using standardised statistical procedures. Gerdessen et al. [7] showed that using Mixed Integer Linear Programming makes the development process faster, more standardised and transparent. However, both systems still require an expert to run them, and the developed questionnaires need validation. The use of machine learning seems an excellent option to work with existing, already validated questionnaires and adapt them to different needs and not have an expert involved to design the questionnaires by hand, but rather use expert knowledge to supervise and validate the machine-learning outcomes. However, to the best of our knowledge, machine learning has mainly been used just to estimate nutrient intake or to detect dietary patterns [8,9,10]. Dimensionality reduction methods such as Principal Component Analysis (PCA) and Hierarchical Cluster Analysis (HCA) have been used in order to detect correlations between different food groups [11] also involving data gathered from FFQs [12]. Although not used in such a way, this approach could also be used when reducing the number of questions from existing questionnaires.

In our previous work, we explored a few approaches that could help automate the development of FFQs and adjust the questionnaires to the users’ needs. So far, we have worked on single-target problems, trying to predict only one nutrition aspect at a time. First, we explored the ranking of questions in FFQs [13], which can help gather the most critical questions in a specific order that delivers the best possible prediction accuracy with each added question. Next, we compared different dimensionality reduction algorithms [14,15] that help shorten extremely long FFQ without losing too much information. Therefore, FFQs are more user-friendly and easier to implement in mobile applications for self-monitoring and self-assessment.

In this paper, we explore different approaches to reduce the number of questions shown on the screen in the LBC mobile application. We consider the problem of predicting the quality of several nutrition aspects from the questionnaire as a multi-target regression problem, which means we predict several values simultaneously [16,17]. We propose a method that uses the distance from predicted values to threshold values (in other words, how much the user underachieves their goals) as weights, considers the users’ answers and consequently adjusts the subset of questions to those most important for the most problematic goals. In Section 2, we describe the questionnaire used in this paper, explain the experimental setup and propose the function that calculates the distance to the threshold values and transforms them into weights. The results are presented in Section 3 and discussed in Section 4. There, we also discuss the limitations of the study. We conclude the paper in Section 5 with a summary and plans for future work.

## 2. Materials and Methods

### 2.1. FFQ and Dataset

The FFQ used in this paper follows the principles of the Mediterranean diet and is currently implemented in the LBC application [18] developed by the Fondanzione Bruno Kessler as a part of the national program TrentionSalute [19] in the Trentino region, Italy.

**Figure 1:**
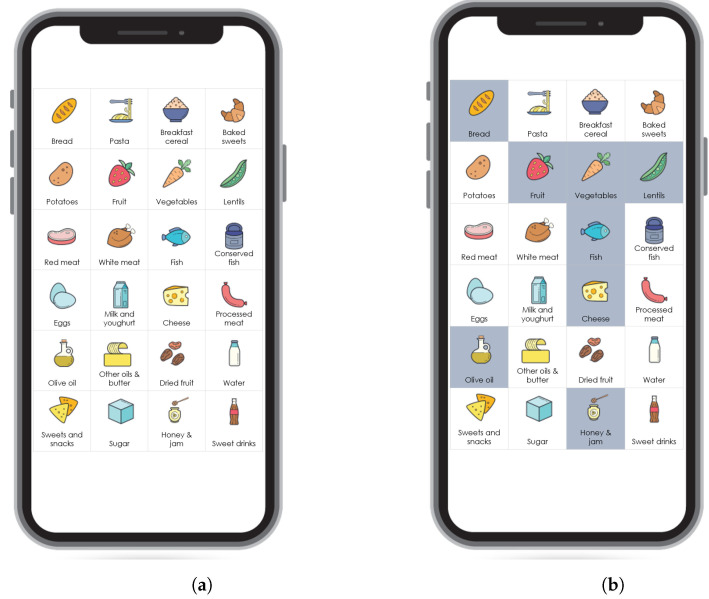
Example of FFQ representation as implemented in the LBC application. The FFQ is represented as a grid of images, and the system asks the user about his daily consumption of the 24 food items. The number of button presses on each image indicates the number of consumed portions of the food item presented with the corresponding image/question. (**a**) The figure on the left represents the FFQ in which 24 questions are represented in a 6 × 4 grid. (**b**) The figure on the right represents an example of a filled-in questionnaire after the user has marked the consumption of at least one portion of bread, fruit, vegetables, lentils, fish, cheese, olive oil and honey. The chosen food items have a darker (gray) background.

The questionnaire asks the user about the daily consumption of 24 food items: bread, pasta, breakfast cereals, baked sweets, potatoes, fruit, vegetables, red meat, white meat, fish, conserved fish, eggs, lentils, milk/yoghurt, cheese, olive oil, other oils/butter, dried fruit, water, processed meat, sweets/snacks, sugar, honey/marmalade and sweet drinks intake. The outcomes of the questionnaire are quality scores of eating habits regarding 11 nutrition aspects: fruit and vegetables, carbohydrates, proteins, milk, oil, water, dried fruit, processed meats, sweets/snacks, sweet drinks, and sugar. The questionnaire is presented with a grid of images (buttons), and the system asks the user about his daily consumption of the 24 food items. The number of button presses on each image indicates the number of consumed portions of the food item presented with the corresponding image/question (Figure 1). For simplicity, we use the expression *questions* for food items from this point on.

While implementing the FFQ in the LBC application offers a good user experience, it also makes it easy to provide partial responses—the users may answer only the questions they deem important. This is not a problem for the users, as the application is intended for self-monitoring, and they can use it as they see fit, but the quality of the data collected with the application was low. Since the method proposed in this paper is general and can be used on any population without particularly unusual dietary habits, we thus used a higher-quality dataset to develop and test it. The questionnaire in the SiMenu had 104 questions, asking the users about the frequency of consumption of different food items. As the questions included in the LBC application questionnaire are a direct subset of the questions in the SiMenu questionnaire, the transformation of the answers to fit the LBC application questionnaire was very straightforward.

### 2.2. Methods

#### 2.2.1. Problem Outline

FFQ representation using a grid of images to list questions is user-friendly and allows a more interactive way of self-reporting dietary habits. However, although the number of questions is only 24 and the questionnaire is not very extensive, the mobile application screen still seems crowded, slightly overwhelming and challenging to read for some users, especially elderly ones. Therefore, our task was to find a way to reduce the list of questions and use machine learning to predict the quality of achieving goals that the user has activated (decided to follow) from the 11 goals listed in Section 2.1.

The main idea was to find the subset of questions that would allow the algorithm to highlight more problematic goals from those the user chose to follow (activated goals). Users usually have better dietary habits regarding some goals than others. For example, a user might have decided to use the application to self-monitor his vegetable, protein and sugar consumption habits. In his meals, he usually includes a portion of vegetables and a portion of fish or meat. He always finishes his lunch and dinner with a dessert. When comparing his quality scores to the optimal amounts of vegetables, protein and sweets, the algorithm will detect that the user eats enough protein and should maybe include more vegetables, but he overeats sugar. We wanted to teach our algorithm to pay more attention to the problematic goals. The most attention should go to sugar consumption, a little less (but still some) attention should go to the goal regarding vegetable intake, and the least attention should go to protein intake (as it is the best). This is included in the algorithm as weights assigned to goals and helps better assess the more problematic goals, which can then be used to give better recommendations or identify the important questions for the user.

In this section, we first describe the pipeline used in the experiments. Next, we describe the machine-learning techniques used in the experimental setup. Finally, we describe a function that personalises the questions’ importance by transforming distance to the optimal quality scores for the activated goals into weights. With this, the application can determine the most informative questions based on the user’s previous answers.

#### 2.2.2. Experimental Setup

When reducing the number of questions, we strove to keep half the questions at most and explored options where we tried to predict targets by using 4, 6, 9 or 12 questions. These numbers are chosen based on the mobile application layout (Figure 1). We want to keep the image grid representation of the questionnaire for better user experience, and visually pleasing options are to show a 2×2 grid, 3×2 grid, 3×3 grid or 4×3 grid.

**Figure 2:**
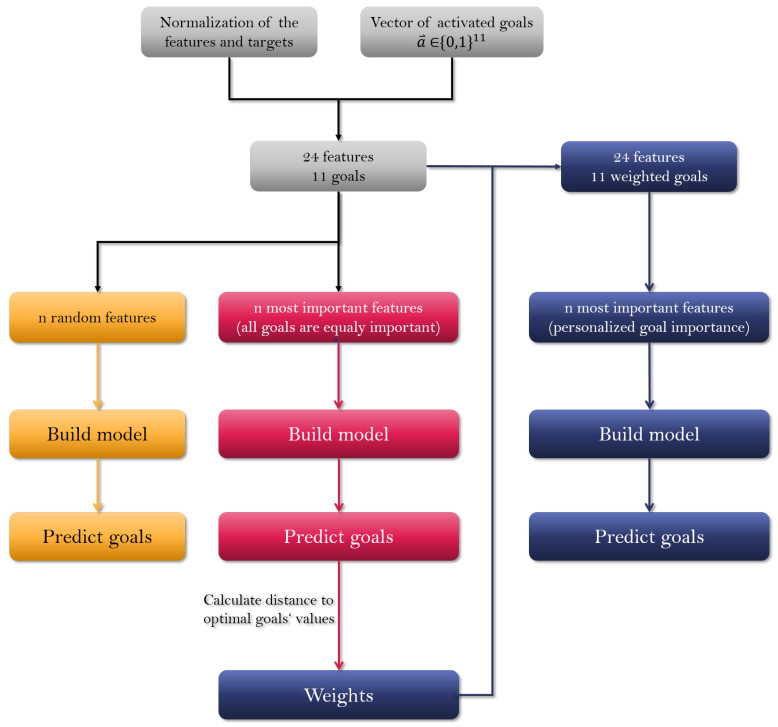
Graphical representation of the pipeline. We conduct three types of experiments: (1) we choose *n* random features (yellow section); (2) we choose *n* best static features in the case where all chosen goals are equally important (pink section); (3) we find *n* most important features based on previous answers gathered from the user (blue section).

We conducted three different experiments to reduce the number of questions: (1) the subsets of questions were chosen randomly; (2) we chose the most important questions by calculating their importance as features for predicting nutrition quality scores assuming all activated goals are equally important; (3) we calculated the distance of predicted quality scores’ values from the experiment to optimal quality scores for activated goals only, transformed them to weights, and recalculated feature importance (Figure 2).

For the first experiment that was used as a baseline, we decided to choose questions randomly. Another option would be to use one of the dimensionality reduction methods such as Pearson correlation analysis [20], as used in our previous work [14], or PCA, which is sometimes used for multivariate analysis of relations between food groups [11,12]. However, although all of the mentioned methods are potent tools, they rely only on correlations between the features (questions) and do not consider the chosen goals. Moreover, in our initial experiments, we did perform PCA and discovered that the first six principal components are built from just five of the original questions, while the seventh principal component is built from all of the questions, which would not reduce the number of questions. Combining this finding with the fact that Pearson correlation, PCA and HCA do not consider target variables and that the questionnaire we were dealing with is very simple indicated that some of the goals would never be predicted well, as the questions related to them would never be included in the image grid. Therefore, choosing random questions gives some goals at least a fighting chance.

#### 2.2.3. Multi-Target Regression and Feature Selection

We conducted the experiments as multi-target regression problems, which means that several quality scores are predicted simultaneously [21]. In our previous work, we already explored a single-target prediction of quality scores. We explored question ranking in FFQs [13] and compared different dimension reduction algorithms [14,15]. Based on the conclusions from our previous work, we decided to use linear regression as the prediction model since the features and targets indeed have a linear relationship, and more complex machine-learning models, such as Random Forest or XGBoost, tend to overfit.

In the LBC application, users are free to activate (follow) from 1 to 11 goals. Therefore, the case in which the user chooses just one goal can be treated as a single-target problem, and in this case, experiment (3) does not differ from the experiment (2), which was already addressed in our previous work [14,15]. Therefore, this paper focuses on cases where the user chooses at least two goals.

Answers to the questions are first transformed into a ‘portion per day’ measure. A similar technique is used with the goals. In machine learning, the transformed answers to the questions are called features, and the quality scores are called targets. Both features and targets are then scaled using the min–max normalisation:

$$x^{\prime }=\frac{x-\min(x)}{\max(x)-\min(x)}$$

which scales all values to the interval [0,1].

For machine learning, we first split the dataset into two parts—train and test set in a 3:1 ratio. From there on, we conducted three different experiments (Figure 2):

1. *Random features.* We choose n random features, n being a number from a set of {4,6,9,12}, build the model on the train set and test it on the test set.
2. *Statistically optimised features.* We choose the best *n* features, n being a number from a set of {4,6,9,12}. Feature selection is then performed on the train set using 5-fold cross-validation. Then, by using an integrated function in the sklearn library, feature importance is calculated on each fold and then averaged over the folds. The chosen features are then used to build a model on the train set, and the evaluation of the model is completed on the test set. This experiment considers all of the goals equally important for the user.
3. *Personalised features.* The third experiment is split into two steps. The first step is the same as the second experiment. The second step takes the predicted values from the second experiment and calculates the distance to the optimal values of the goals (see the following Section 2.2.4 for details). The distances are transformed into weights by being scaled to the interval [0,1]. The vector of weights is then used to scale the targets (goals), which allows us to emphasise the goals that were further away from the optimal values. We again calculate feature importance. Due to weights, the quality scores are not scaled on the same interval anymore; therefore, the quality scores with higher values are considered more important by the algorithm. This helps us to predict the quality scores of the problematic goals better. Such predictions can be obtained when the user responds to the FFQ for the second time. However, since the LBC application is intended for regular diet monitoring, the user is expected to provide answers periodically. We again choose n features, n being a number from a set of {4,6,9,12}, build the models on the train set and evaluate them on the test set.

#### 2.2.4. Distance to Threshold

The LBC application provides the questionnaire and also the threshold quality scores’ values that have to be reached for some of the goals (vegetable and fruit intake, fish intake, lentils, oil, water intake, dried fruit) or should not be over-reached (carbohydrates, proteins, milk/yoghurt, cheese, processed meats, snacks/sweets, sweet drinks, sugar). We calculate the distance as

$$\mathrm{dist}\left(y_{pred},y_{opt}\right)=\max\left(\left(y_{pred}-y_{opt}\right)*y_{adj},0\right),$$

where $y_{pred}$ are the predicted values, $y_{opt}$ is the vector of threshold values and $y_{adj}$ is the vector adjustments. Values in $y_{adj}$ are either −1 or 1, where 1 stands for *at most* and −1 stands for *at least*. We take only the distances where the difference $\left(y_{pred}-y_{opt}\right)*y_{adj}$ is a positive value, which means that the user does not reach the *at least* value or overreaches the *at most* value. The distances are then scaled to fit the [0,1] interval and used as weights.

## 3. Results

We conducted three experiments: random features, statically optimised features, and personalised features. Additionally, we compared cases when we showed 4, 6, 9 or 12 questions on the image grid.

There are 2037 combinations of goals in which at least two goals are activated. We present the results averaged by the number of activated goals, which is completed for all cases of selected features. This section presents the main results, and the remaining can be found in Appendix A.

**Figure 3:**
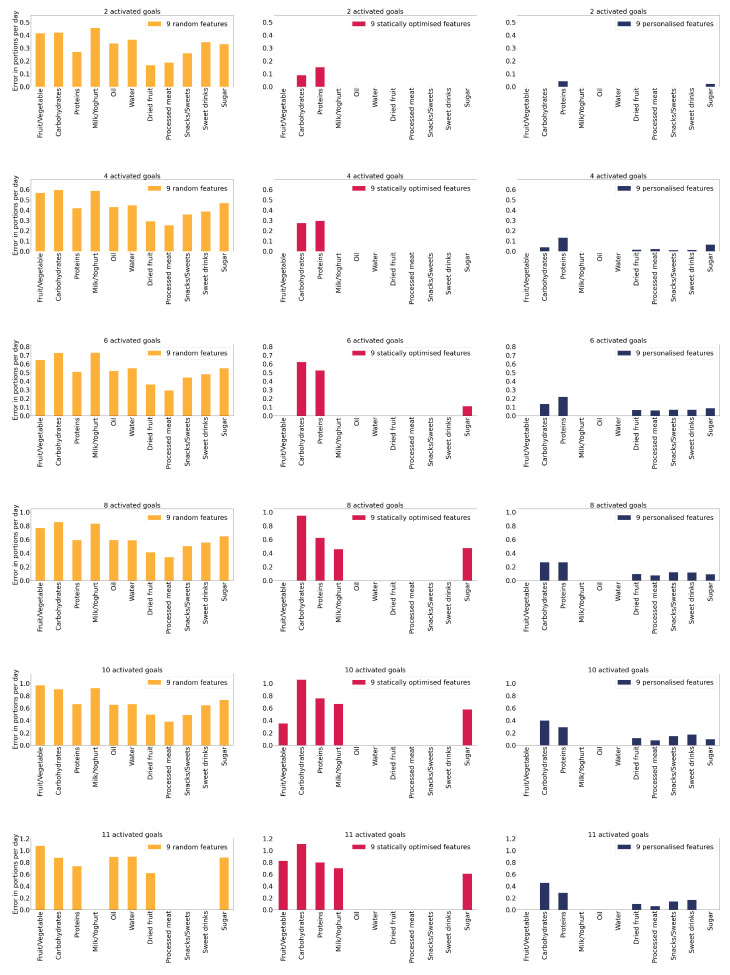
Error in predicting consumed portions for the 11 goals. Each row presents the results for a certain number of activated goals. The first column (yellow bar plots) shows results for random features. The second column (pink bar plots) shows results for statically optimised features. Finally, the third column (blue bar plots) shows the results for personalised features.

In Figure 3, we present a subset of results in which the FFQ comprises nine features. We compare the three experiments (following the colour scheme from Figure 2). Each row represents a different number of activated goals. The results for other numbers of features and the missing numbers of activated goals can be found in Appendix A.

**Figure 4:**
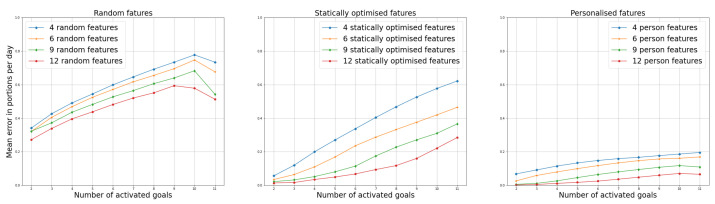
Error in predicted portions per day averaged over the 11 goals. We compare the errors based on the number of activated goals (*x*-axes in the subplots), based on the number of features we want to select (different lines within one subplot), and based on the method for feature selection, each of the three subplots corresponding to one of the methods.

Figure 4 shows aggregated results—we observe how the average error changes with the number of activated goals and the number of selected features.

## 4. Discussion

The LBC application integrates a Food Frequency Questionnaire in a rather innovative way. However, although the questionnaire is reasonably short (24 questions), the representation with the image grid still becomes quite hard to read, especially for elderly users. Therefore, in this paper, we investigated a way to reduce the number of questions. We compared three approaches. Two of them are pretty straightforward. The first one serves as a baseline and simply picks *n* random equations. The second one uses feature selection to determine *n* best features (which correspond to questions), *n* being a number from the set of {4,6,9,12}. Finally, we proposed a method that uses the users’ answers, readjusts the list of most important questions, and better predicts the more problematic goals.

**Figure A1:**
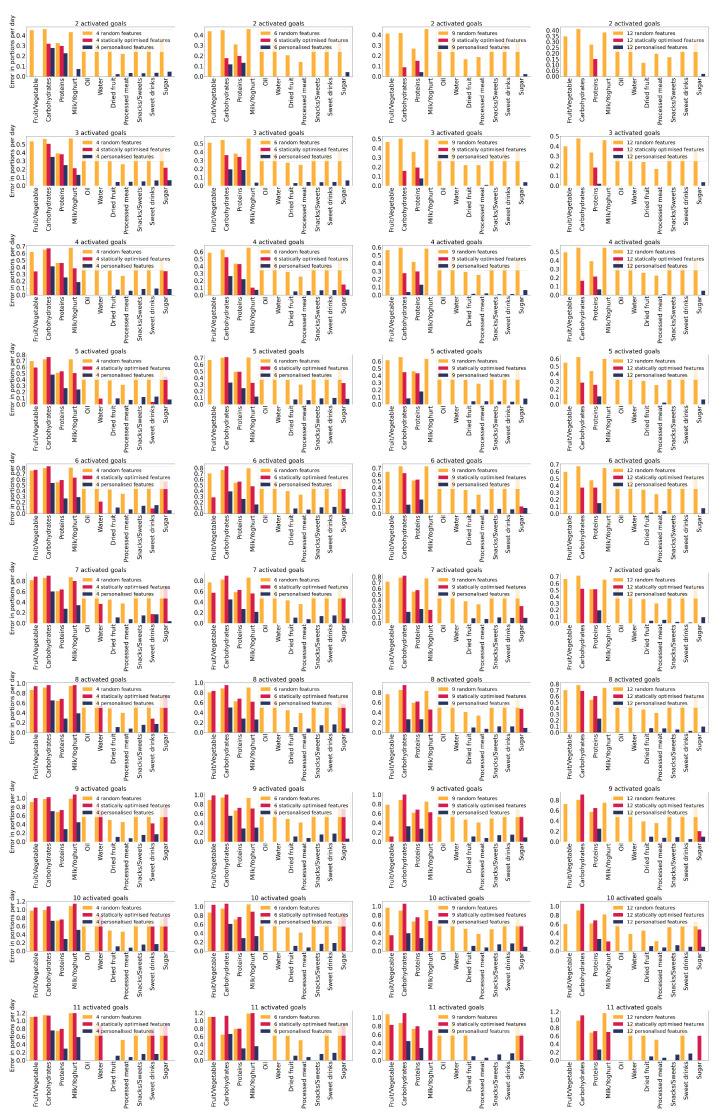
Complete results. We compare results by the number of activated goals (rows), by the number of selected features (columns) and by the method used for feature selection (yellow, pink and blue colours within one subplot).

Based on the results in Figure 3 and Figure 4, one can conclude that the suggested algorithm helps the application predict the problematic goals with a lower error. Therefore, it appears to be a better approach than reducing the questions to those selected by feature selection under the assumption that all the goals are equally important. Furthermore, while Figure 3 only presents a case where we choose nine features, similar trends appear with other examples, as shown in Figure A1.

Another conclusion from the experiments is that the prediction error increases with more activated goals. It also decreases with the number of selected features. Based on these conclusions, nine or 12 features or questions would be the most reasonable choice to be implemented in the application.

Another observation regarding Figure 3 is that statically optimised features have more significant prediction errors and personalised features have more but more minor prediction errors. The explanation is that the questions are spread between all goals with equal importance by finding statically optimised features. With personalised features, more emphasis is given to problematic goals, which means that the prediction for less important goals could become less accurate but still acceptable. The users’ quality score is still close enough to the threshold that the application is not alerted by this fact. In practice, it is better to make a small prediction error with the goals recognised as less problematic and emphasise goals where the user achieves a worse quality score, as these are the goals the user needs to improve first. Once the problematic goals are improved, the weights become redistributed, and the user can also focus on improving other goals.

### Limitations

We conducted our experiments on a dataset derived from a dataset from the SImenu study [22]. While it would be better to gather data through the LBC application questionnaire, preferably at multiple time points for each user, our dataset is valid for developing algorithms and running experiments. We only need some caution in interpreting the results since they reflect SImenu users more than LBC application users. The questionnaire is also straightforward, and some goals only depend on one question. Therefore, the improvement that more complex methods can bring on is limited. However, based on the experiments’ results, the proposed method could reduce the application questionnaire.

## 5. Conclusions

In this paper, we proposed a method to reduce the number of questions and FFQ used in the LBC application. The number of questions is reduced based on users’ chosen nutrition goals and how well they achieve them. Additionally, the application should give more attention to the more problematic goals related to nutrition habits requiring the most improvement.

We compared three methods for choosing the questions in the FFQ—*random features*, *statically optimised features* and *personalised features*. We considered cases where the application shows 4, 5, 9 or 12 questions instead of 24. We concluded that the proposed method—*personalised features*, which considers that goals are not equally essential and considers users’ previous answers, performs the best for the task. In our future work regarding this particular FFQ, we would like to explore further the possibilities of adopting the questionnaire to the users’ preferences based on their answers. We would also like to integrate the proposed method into the existing application and test it in the field on the actual longitudinal data.

In addition to working with this particular questionnaire, we plan to test the proposed on more complex questionnaires in which questions and goals are more intertwined than in the FFQ used in this paper and on questionnaires where questions are asked more conventionally: question by question. Finally, we would like to explore more options to personalise the questionnaires based on users’ previous answers by using active learning [23]—where the user’s last answer to the question could be used to re-evaluate the importance of the remaining questions, and the next most important question is asked next.

## Data Availability

The data used in this study are available in anonymous form from the corresponding author upon motivated reasonable request.

## Abbreviations

The following abbreviations are used in this manuscript:

FFQ	Food Frequency Questionnaire
LBC	Lifestyle Behaviour Change
PCA	Principal Component Analysis
HCA	Hierarchical Cluster Analysis
